# The Legal Status of the Hospital Ship in War-Time

**Published:** 1915-04-17

**Authors:** 


### THE LEGAL STATUS OF THE HOSPITAL SHIP IN WAR-TIME.

The international rules as to the care of the sick and wounded in war have been already explained in our pages; and, as to these, land and sea warfare are, *mutatis mutandis*, fairly similar. But the hospital ship being peculiar to naval operations, a brief statement of the recognised laws regarding it may be of interest and perhaps of practical use. For a more detailed account of the question reference is made to Convention 10 of the Hague Conference, 1907, by which the original Geneva Rules of 1864 were modernised.

Hospital ships are of two classes, either military hospital ships—that is, ships specially assigned by a belligerent State to aid the wounded sick and shipwrecked; or ships equipped by philanthropic private individuals or societies which are placed by them at the disposal and under the control of one of the belligerents, and the names of which have been notified to the other belligerent. The military hospital ship is distinguished by being painted white outside with a broad horizontal green band, while the other hospital ships are painted white outside with a horizontal red band. The boats of hospital ships must also be respectively distinguished by similar painting. All hospital ships must make themselves known by hoisting with their national flag the Red Cross flag, and if they wish to ensure by night the freedom from interference to which they are entitled, they should take the necessary measures to make their flag and special painting sufficiently plain; but this is subject to the assent of the belligerent whom they are accompanying, so that, if for strategic reasons lights are not shown, the hospital ship loses its immunity from attack, as would of course also happen if a hospital ship were made use of to carry out and commit acts hostile to the other belligerent.

Indeed to take such active part in war as has just been suggested would deservedly forfeit a hospital ship's privileges, for the belligerents are held to undertake that hospital ships are not to be used for any military purpose, and must not in any way hamper the movements of the combatants. Their duty is to afford relief and assistance to the wounded sick and shipwrecked of the belligerents without distinction of nationality. And hospital ships engaged on that duty and observing the conditions of their service are to be respected, and cannot be captured.

To ensure that hospital ships are not made use of for purposes of active warfare, they are subject to the right of search which may be exercised by any belligerent. The necessity for this is illustrated by a case which occurred during the Russo-Japanese war, when the Japanese fleet being suspicious of a Russian hospital ship captured it, and the matter being subsequently brought before a Prize Court, it was found (1) that the hospital ship had communicated the orders of the Russian Commander-in-Chief to other vessels; (2) had been instructed to purchase war material; and generally (3) that there was reasonable evidence justifying the conclusion that it had been constantly employed for military purposes. The ship was therefore condemned as prize, and its complement made prisoners of war.

The foregoing remarks relate to hospital ships only, and do not concern the hospital staff of other ships, the rule as to which is that the medical and hospital staff of *any captured ship* is inviolable, and its members cannot be made prisoners of war. On leaving the ship they take with them the objects and surgical instruments which are their own private property; and while in the control of their captors they must discharge their usual duties if necessary, receiving from the captor the same allowances and pay as are granted to persons of the same rank in his own navy.

